# Prevalence of Polycystic Ovary Syndrome in Patients With Pediatric Type 2 Diabetes: A Systematic Review and Meta-analysis

**DOI:** 10.1001/jamanetworkopen.2021.47454

**Published:** 2022-02-15

**Authors:** Milena Cioana, Jiawen Deng, Ajantha Nadarajah, Maggie Hou, Yuan Qiu, Sondra Song Jie Chen, Angelica Rivas, Laura Banfield, Haifa Alfaraidi, Ahlam Alotaibi, Lehana Thabane, M. Constantine Samaan

**Affiliations:** 1 Department of Pediatrics, McMaster University, Hamilton, Ontario, Canada; 2 Division of Pediatric Endocrinology, McMaster Children’s Hospital, Hamilton, Ontario, Canada; 3 Michael G. De Groote School of Medicine, McMaster University, Hamilton, Ontario, Canada; 4 Health Sciences Library, McMaster University, Hamilton, Ontario, Canada; 5 College of Medicine, King Saud bin Abdulaziz University for Health Sciences, Division of Endocrinology, Department of Pediatrics, Ministry of the National Guard Health Affairs, Riyadh, Saudi Arabia; 6 Department of Pediatrics, Division of Pediatric Endocrinology, King Abdullah bin Abdulaziz University Hospital, Princess Noura University, Riyadh, Saudi Arabia; 7 Department of Health Research Methods, Evidence and Impact, McMaster University, Hamilton, Ontario, Canada; 8 Department of Anesthesia, McMaster University, Hamilton, Ontario, Canada; 9 Centre for Evaluation of Medicines, St Joseph’s Health Care, Hamilton, Ontario, Canada; 10 Biostatistics Unit, St Joseph’s Healthcare, Hamilton, Ontario, Canada

## Abstract

**Importance:**

The prevalence of pediatric type 2 diabetes (T2D) is increasing globally. Girls with T2D are at risk of developing polycystic ovary syndrome (PCOS), but the prevalence of PCOS among girls with T2D is unknown.

**Objective:**

To determine the prevalence of PCOS in girls with T2D and to assess the association of obesity and race with this prevalence.

**Data Sources:**

In this systematic review and meta-analysis, MEDLINE, Embase, CINAHL, Cochrane Central Register of Controlled Trials, Cochrane Database of Systematic Reviews, Web of Science: Conference Proceedings Citation Index–Science, and the gray literature were searched from inception to April 4, 2021.

**Study Selection:**

Two reviewers independently screened for studies with observational study design that recruited 10 or more participants and reported the prevalence of PCOS in girls with T2D.

**Data Extraction and Synthesis:**

Risk of bias was evaluated using a validated tool, and level of evidence was assessed using the Oxford Centre for Evidence-Based Medicine criteria. A random-effects meta-analysis was performed. This study follows the Meta-analysis of Observational Studies in Epidemiology (MOOSE) reporting guideline.

**Main Outcomes and Measures:**

The main outcome of this systematic review was the prevalence of PCOS in girls with T2D. Secondary outcomes included assessing the associations of obesity and race with PCOS prevalence.

**Results:**

Of 722 screened studies, 6 studies involving 470 girls with T2D (mean age at diagnosis, 12.9-16.1 years) met the inclusion criteria. The prevalence (weighted percentage) of PCOS was 19.58% (95% CI, 12.02%-27.14%; *I*^2^ = 74%; *P* = .002). Heterogeneity was moderate to high; however, it was significantly reduced after excluding studies that did not report PCOS diagnostic criteria, leading to a calculated prevalence (weighted percentage) of 24.04% (95% CI, 15.07%-33.01%; *I^2^* = 0%; *P* = .92). Associations with obesity and race could not be determined because of data paucity.

**Conclusions and Relevance:**

In this meta-analysis, approximately 1 in 5 girls with T2D had PCOS, but the results of this meta-analysis should be considered with caution because studies including the larger numbers of girls did not report the criteria used to diagnose PCOS, which is a challenge during adolescence. The associations of obesity and race with PCOS prevalence among girls with T2D need further evaluation to help define at-risk subgroups and implement early assessment and treatment strategies to improve management of this T2D-related comorbidity.

## Key Points

**Question:**

What is the prevalence of polycystic ovary syndrome (PCOS) among adolescents with type 2 diabetes (T2D)?

**Findings:**

In this systematic review and meta-analysis involving 470 girls across 6 studies, the prevalence of PCOS was 19.58%, a prevalence that is substantially higher than that of PCOS in the general adolescent population.

**Meaning:**

These findings suggest that PCOS is a common morbidity in girls with T2D, and it is critical that active screening for PCOS in girls with T2D is initiated at diabetes diagnosis and follows international evidence-based guidelines for diagnosing PCOS in adolescents.

## Teaser

This systematic review and meta-analysis aims to determine the prevalence of polycystic ovary syndrome in girls with type 2 diabetes and to assess the association of obesity and race with this prevalence.

## Introduction

Over the past 3 decades, type 2 diabetes (T2D) has made the transition from being an adult disease to being a pediatric disorder.^1,2,3,4,5,6,7,8^ T2D in youth is an aggressive disease with multiple associated comorbidities and poor response to current therapies; it is also associated with higher morbidity and mortality rates than adult-onset T2D.^9,10,11^

Polycystic ovary syndrome (PCOS) is a complex endocrine disorder that occurs in 1.14% to 11.04% of adolescent girls globally.^12,13^ The diagnostic criteria for PCOS during adolescence include the combination of menstrual irregularities according to time since menarche and clinical or biochemical hyperandrogenism after excluding other possible causes.^14,15,16,17,18^ Pelvic ultrasonography is not recommended for PCOS diagnosis in girls who are less than 8 years since menarche according to international evidence-based guidelines,^18^ because it is associated with overdiagnosis of PCOS.^19^ Insulin resistance and compensatory hyperinsulinemia are present in 44% to 70% of women with PCOS,^20,21^ suggesting that they are more likely to develop T2D.^22,23,24^

PCOS is also associated with a range of cardiometabolic diseases, including hypertension and dyslipidemia, as well as mental health disorders and future infertility.^25,26,27,28^ Importantly, girls with T2D and PCOS are at an increased risk of depression.^29^ However, although PCOS is associated with a range of conditions that are related to obesity, the association of PCOS with obesity is not well understood. PCOS is more common in adolescents with obesity,^12^ yet insulin resistance is at times present in patients with PCOS regardless of their body mass index (BMI).^30,31,32^

In addition, pediatric T2D disproportionately affects female patients, and its rates are increased among minoritized racial and ethnic groups.^1,2,4,5^ Determining the scale of PCOS in T2D and the association of obesity and race with PCOS genesis can inform personalized screening and treatment strategies in this population.^33,34^ The objectives of this systematic review and meta-analysis were to determine the prevalence of PCOS in girls with T2D and to assess the association of obesity and race with PCOS prevalence.

## Methods

### Systematic Review Protocol and Registration

This systematic review and meta-analysis has been registered with PROSPERO.^35^ Institutional review board approval and informed consent were not sought because the data were anonymous and publicly available, in accordance with 45 CFR §46. The manuscript was developed and reported in accordance with the Meta-analysis of Observational Studies in Epidemiology (MOOSE) reporting guideline.^36^

### Search Strategy and Eligibility Criteria

Searches in MEDLINE, Embase, CINAHL, Cochrane Central Register of Controlled Trials, and Cochrane Database of Systematic Reviews were developed by a senior health sciences librarian (L.B.) (eTable 1, eTable 2, eTable 3, and eTable 4 in the Supplement). Gray literature searches were conducted in ClinicalTrials.gov, Cochrane Central Registry of Controlled Trials, and Web of Science Conference Proceedings Citation Index–Science (eTable 5 in the Supplement). In addition, we searched the reference lists of eligible articles at the full-text screening stage for additional papers that fulfill the inclusion criteria.

The databases were initially searched from inception to February 4, 2019, and updated searches were run on February 20, 2020, and April 4, 2021. There were no language restrictions, but searches were limited to human studies. Terms for pediatrics and T2D were combined with language referencing PCOS, prevalence, and observational study design. Where a conference abstract was considered for inclusion, we searched the databases for a full-text publication and contacted the principal investigator if the publication could not be located.

Studies were included if they reported PCOS in girls diagnosed with T2D at age 18 years or younger. The studies included cross-sectional, retrospective, and prospective cohort studies, with a sample size of 10 or more patients, which reported the prevalence of PCOS in patients with T2D. We included all studies reporting on PCOS regardless of whether PCOS definition was reported.

The exclusion criteria included studies of patients with gestational diabetes. When encountering studies with serial reporting of data, we planned to include the report with the largest sample size.

### Study Selection, Data Abstraction, and Quality Appraisal

Title, abstract, and full-text screening, data abstraction, risk of bias, and level of evidence assessments were performed by 2 independent reviewers in 3 teams (M.C., A.N., M.H., Y.Q., S.S.J.C., and A.J.R). Disagreements were resolved through discussion, or by a third reviewer (M.C.S.) if they persisted.

Data abstractions were done using a standardized form. We collected data including study title, author name, publication year, country, study design, age at diabetes diagnosis, age at study participation, duration of diabetes, sample size, and prevalence of obesity in participants, where available. We also extracted data on PCOS definition and total and race-based prevalence of PCOS. We contacted the principal investigators to collect any missing data.

Risk of bias was evaluated using a validated tool for prevalence studies.^37^ The tool assesses the internal and external validity of the studies, rating overall risk of bias as low (score >8), moderate (score 6-8), or high (score ≤5).

Level of evidence was assessed using the Oxford Centre for Evidence-Based Medicine criteria.^38^ The scale rates the appropriateness of each study to answer the research question, taking into account study design, study quality, imprecision, indirectness, and inconsistency.^38^

### Statistical Analysis

We performed a meta-analysis using a random-effects model when 2 or more studies reporting the prevalence of PCOS used similar design, methods, and populations.^39,40^ If studies could not be included in the meta-analysis, the results were reported as a narrative summary and tabulated. Prevalence values were calculated using raw proportions of the number of girls with PCOS and T2D divided by the total number of girls diagnosed with T2D. Study weights were calculated from the inverse of the variance of prevalence value. All studies were then pooled according to weight, and a pooled prevalence value was determined. Because no studies had prevalence values close to 0% or 100%, we did not use transformations in our calculations.^40^

The primary outcome for this review was the pooled prevalence of PCOS with a 95% CI. Both inconsistency index (*I*^2^) and χ^2^ test *P *values were used to quantify heterogeneity, with *I*^2 ^> 75% and *P* < .10 considered as significant cutoffs for heterogeneity.^41^

We had originally planned to perform subgroup analyses by race if 10 or more studies were included in the meta-analysis. However, these analyses could not be completed because of the limited number of eligible studies. Because of the number of included studies that did not report PCOS diagnostic criteria, we also conducted a post hoc sensitivity analysis excluding these studies to examine their impact on prevalence and heterogeneity. The meta-analysis was conducted using the metafor package in RStudio statistical software version 1.1.383 and R statistical software version 3.4.3 (R Project for Statistical Computing).^42,43,44^

## Results

### Study Selection

**Figure 1.**
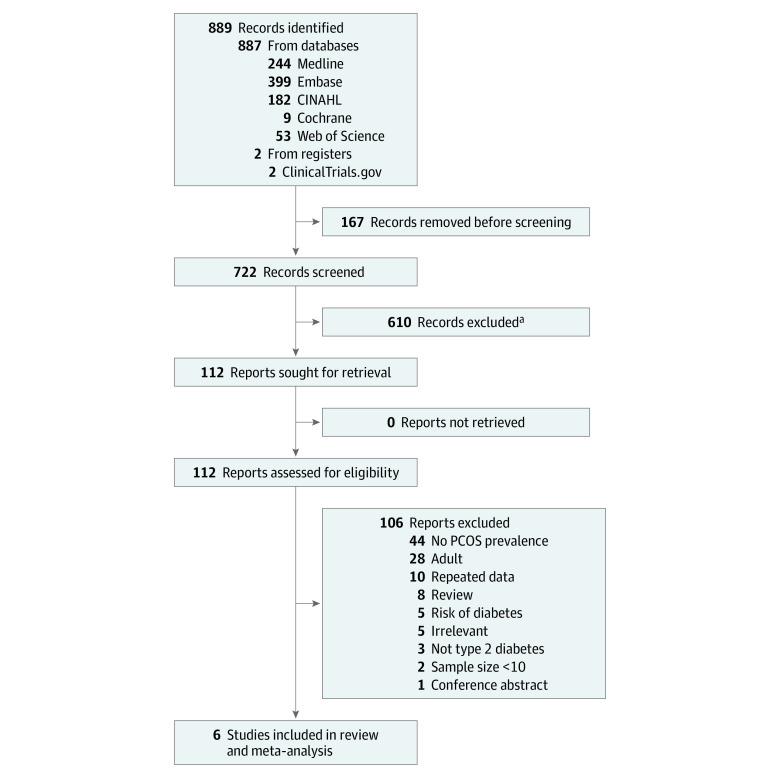
Study Flow Diagram PCOS indicates polycystic ovary syndrome. ^a^The exclusion criteria included studies of patients with gestational diabetes.

Of 722 screened articles, 6 studies^45,46,47,48,49,50^ involving 470 girls met our inclusion criteria (Figure 1). The Table reports the characteristics of the included studies. Five were retrospective cohort studies,^45,46,47,48,49^ and 1 was a prospective cohort study.^50^ The mean (SD) age at diagnosis of T2D ranged from 12.9 to 16.1 years, and the mean duration of T2D ranged from inclusion at diagnosis of T2D to 5.9 years after diagnosis.

**Table.** Characteristics of Included Studies

Study	Design	Mean (SD) or mean (95% CI), y			PCOS prevalence, No. (%)	Sample size, No.	Patients, No. (%)			Risk of bias	Level of evidence	Method of PCOS assessment	US use
		Age at T2D diagnosis	Age at study enrollment/measurement	Duration of T2D			Ethnic distribution	Prevalence of PCOS by ethnic group	Prevalence of obesity in female patients				
Amed et al,^45^ 2012, Canada	RC	Indigenous: 12.9 (12.4-13.4)^a^	Indigenous: 12.9 (12.4-13.4)^a^	0	11 (8.5)	130	White 36 (27.7)	White: 6 (17)	NR	Moderate	3	Medical records	No
		White: 14.4 (13.8-15.1)^a^	White: 14.4 (13.8-15.1)^a^				Indigenous: 64 (49.2)	Indigenous: 1 (2)					
		Other ethnicity: 14.3 (13.7-14.9)^a^^,^^b^	Other ethnicity: 14.3 (13.7-14.9)^a^^,^^b^				Other: 30 (23.1)^b^	Other: 5 (17)^,^^b^					
Amutha et al,^46^ 2012, India	RC	16.1 (2.5)^a^^,^^c^	22.2 (9.7)^a^^,^^c^	5.94 (0.48)^a^^,^^c^	45 (23.1)	195	Indian: 195 (100)^d^	Indian: 45 (23.1)^d^	NR	Moderate	1	Clinical symptoms (unspecified)	No
Balasanthiran et al,^47^ 2012, UK	RC	15.2 (3.34)^a^	21.2 (3.19)^a^	5.4 (3.09)^a^	6 (22)	27	Bangladeshi: 11 (25)	NR	NR	Moderate	2	Hirsutism (documented using the Ferriman-Gallwey score) and menstrual irregularities	No
							Black African: 4 (9)						
							Black Caribbean: 4 (9)						
							Indian: 7 (16)						
							Pakistani: 9 (20)						
							White British: 6 (14)						
							Unclear: 3 (7)^a^						
Pérez-Perdomo et al,^48^ 2005, Puerto Rico	RC	<10 y: 5 (7.9%)	<10 y: 5 (7.8%)	NR	12 (21)	58^e^	NR	NR	NR	High	3	Medical records	No
		10-14 y: 36 (57.1%)	10-14 y: 29 (45.3%)										
		15-18 y: 21 (33.3%)	15-18 y: 29 (45.3%)										
		≥19 y: 1 (1.6%)	≥19 y: 1 (1.6%)										
Zdravkovic et al,^49^ 2004, Canada	RC	13.5 (2.2)^a^	13.5 (2.2)^a^	0	6 (23)	26	African Canadian: 11 (27)	NR	NR	Low	2	History of irregular menstrual periods, obesity, or biochemical evidence of hyperandrogenism	No
							White: 6 (15)						
							First Nations: 1 (2)						
							Hispanic: 4 (10)						
							South or East Asian: 19 (46)^a^						
Shield et al,^50^ 2009, UK and Republic of Ireland	PC	13.6 (9.9-16.8)^a^	14.5 (10.8-17.8)^a^	1	9 (26)	34	Black: 12 (17)	NR	NR	Low	2	Clinical features of PCOS including hirsutism and menstrual disturbance, supported by biochemical evidence of low sex hormone-binding globulin and luteinizing hormone predominance or US	Yes
							Mixed or Chinese: 6 (8)						
							South Asian: 13 (18)						
							White: 42 (57)^a^						

Abbreviations: NR, not reported; PC, prospective cohort; PCOS, polycystic ovary syndrome; RC, retrospective cohort; T2D, type 2 diabetes; US, ultrasonography.

^a^ Value is representative of whole cohort of the study including male patients.

^b^ The study did not specify what is meant by other race or ethnicity.

^c^ Data are mean (SE).

^d^ Ethnic distribution is assumed to match country of origin.

^e^ We subtracted from sample size girls with T2D diagnosed at age less than 10 years because they are unlikely to be pubertal and present with PCOS, and girls with T2D diagnosis at age greater than 18 years because this does not fit our inclusion criteria.

### Prevalence of PCOS

**Figure 2.**
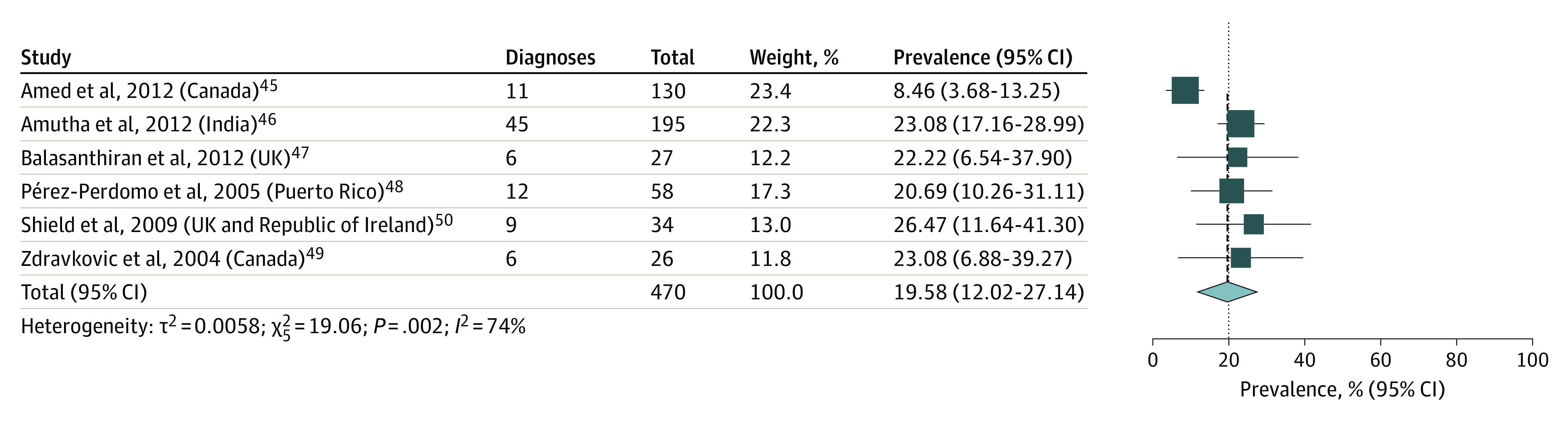
Forest Plot Showing Prevalence of Polycystic Ovary Syndrome in Patients With Pediatric Type 2 Diabetes Size of boxes is proportional to weight of each study. Solid lines represent confidence interval for the prevalence value reported in each study. Dotted line represents the pooled estimate.

The prevalence (weighted percentage) of PCOS across the included studies was 19.58% (95% CI, 12.02%-27.14%) (Figure 2).^45,46,47,48,49,50^ Heterogeneity was moderate to high (*I*^2^ = 74%; *P* = .002).

### Prevalence of PCOS by Diagnostic Criteria

There were variations in the PCOS definition in the included studies. The PCOS diagnostic criteria used in the included studies are summarized in eTable 6 in the Supplement. Common diagnostic criteria across the different guidelines included persistent oligomenorrhea and clinical and/or biochemical hyperandrogenism. Three studies used these criteria to make a PCOS diagnosis (Table).^47,49,50^ The remaining studies reported PCOS diagnosis according to medical records review or clinical symptoms; however, the exact criteria were not defined.^45,46,48^ In addition, none of the studies reported time to menarche, which is important for establishing PCOS diagnosis.^14,15,16,17,18^

**Figure 3.**
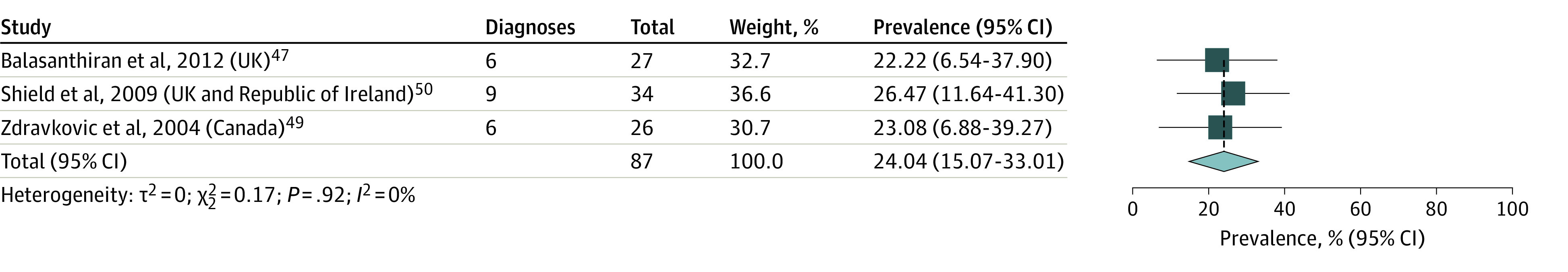
Forest Plot Showing Prevalence of Polycystic Ovary Syndrome (PCOS) in Patients With Pediatric Type 2 Diabetes in Studies Following PCOS Definition in Adolescence Size of boxes is proportional to weight of each study. Solid lines represent confidence interval for the prevalence value reported in each study. Dotted line represents the pooled estimate.

We conducted a sensitivity analysis excluding studies that did not report PCOS diagnostic criteria (87 girls) (Figure 3). The pooled PCOS prevalence (weighted percentage) increased to 24.04% (95% CI, 15.07%-33.01%) with a substantial reduction in heterogeneity (*I^2^* = 0%; *P* = .92).^47,49,50^

### Prevalence of PCOS by Race

Only 2 studies^45,46^ reported the prevalence of PCOS by race. The prevalence was 17.00% in White individuals (36 girls),^45^ 23.10% in Indian individuals (195 girls),^46^ and 2.00% in Indigenous individuals in Canada (64 girls).^45^

### Obesity and PCOS Prevalence

Although we originally aimed to determine the association of obesity and PCOS, none of the included studies provided information on the prevalence of obesity. Thus, the association between PCOS and obesity could not be evaluated.

### Risk of Bias

Two studies had low risk of bias,^49,50^ 3 studies had moderate risk of bias,^45,46,47^ and 1 had high risk of bias (eTable 7 in the Supplement).^48^ In 3 studies,^46,47,49^ the study population was not representative of the national population, and in 3 other studies^45,47,48^ the sampling frame was not representative of the target population. Cases were not selected using random selection or census data in 2 studies.^45,48^ One study^48^ had nonresponse bias and it was unclear what numerator and denominator were used to calculate PCOS prevalence.

In 3 included studies,^45,46,48^ the diagnostic criteria used for PCOS assessment were unclear. In 1 study,^48^ it was not clear whether all patients were assessed for PCOS using the same methods.

### Level of Evidence

Studies had a level of evidence of 1 (1 study),^46^ 2 (3 studies),^47,49,50^ or 3 (2 studies).^45,48^ Level of evidence was rated down for studies that did not use random sampling,^45,48^ and for those that did not have an adequate sample size.^47,49,50^

## Discussion

The prevalence of pediatric T2D is increasing globally, and the majority of these patients are female.^6,7,51^ PCOS is a comorbidity of T2D that is associated with substantial metabolic, cardiovascular, and psychological consequences.^23,26,52,53,54^ Thus, the timely assessment and management of PCOS in this high-risk population is critical.^22^ On the basis of studies with mostly moderate risk of bias, this systematic review and meta-analysis demonstrated that approximately 1 in 5 girls with T2D have PCOS. This figure is substantially higher than PCOS prevalence among the general female adolescent population, which is estimated at 1.14% to 11.04%.^12,13^ There was moderate-to-high heterogeneity in the results of the studies included, although most of the heterogeneity may be attributable to the inclusion of studies that did not clearly report the PCOS diagnostic criteria. The prevalence of PCOS by race was reported in single studies, which precluded generalizability, and the association of obesity with PCOS could not be estimated because of the lack of data.

The association between PCOS and T2D in adults is bidirectional.^55,56,57^ Insulin resistance plays a central role in the pathogenesis of PCOS, and studies in adolescents have shown that girls with PCOS have decreased insulin sensitivity and compensatory hyperinsulinemia.^23,30,58^ Insulin increases the sensitivity of the pituitary gland to hypothalamic gonadotropin-releasing hormone, which, in turn, stimulates the production of luteinizing hormone.^59^ Both insulin and luteinizing hormone act synergistically on the ovarian theca cells to upregulate androgen production,^21,55^ which, in turn, reduces adipose tissue adiponectin secretion and insulin sensitivity and upregulates insulin production.^60^ In addition, insulin increases androgen production within the subcutaneous adipose tissue via the upregulation of the aldo-keto reductase 1C3 activity.^61^

Another mechanism that may lead to both insulin resistance and hyperandrogenism is lipotoxicity.^22^ Increased ovarian exposure to fatty acids can lead to the overproduction of androgens,^62,63^ and the enhanced delivery of fatty acids to nonadipose tissues is key to the development of insulin resistance and T2D.^64^

Although earlier studies suggested that obesity-related insulin resistance and hyperinsulinemia can contribute to PCOS pathogenesis,^15^ insulin resistance in patients with PCOS may be present independently of BMI.^30,31,32^ Obesity seems to increase the risk of PCOS only slightly^65^ and might represent a referral bias for PCOS.^12^ Lipotoxicity is a potential mechanism in the development of both T2D and PCOS, and it can occur independently of obesity.^15,66^ In addition, adipose tissue dysfunction is seen in both PCOS and T2D,^22^ as women with PCOS have larger subcutaneous adipocytes for the same degree of total adiposity and BMI, and adipocyte hypertrophy is strongly correlated with insulin resistance and T2D.^67,68^ Further studies are needed to clarify the association of obesity with PCOS pathogenesis in girls with T2D.

Because of the scarcity of studies reporting race-specific data, we could not address the association of race with PCOS prevalence comprehensively. However, our data demonstrate that Indian girls had the highest prevalence, followed by White girls, and then Indigenous girls in Canada. In a retrospective study^69^ assessing PCOS prevalence in 250 adolescents without T2D, 60 patients were African American (65%) and 24 patients (26%) were White. African American patients had a higher BMI and hemoglobin A1_c_ and less dyslipidemia compared with White individuals.^69^ A systematic review^70^ in adult women reported that Chinese women have the lowest prevalence of PCOS, followed by White, Middle Eastern, and Black women. More studies are needed to evaluate the prevalence of PCOS in girls with T2D across different racial groups to aid the development of personalized screening and management strategies.

It is important for PCOS to be diagnosed early to prevent the development of ensuing complications when untreated. PCOS in adolescence is associated with features of the metabolic syndrome, including hypertension, hyperglycemia, and dyslipidemia.^26^ In addition, adolescents with PCOS have higher prevalence of cardiovascular risk factors,^23^ including higher carotid intima thickness, β stiffness index, and reduced arterial compliance compared with patients with obesity and no PCOS.^54^ Psychiatric comorbidities are also prevalent in PCOS, such as anxiety (18%), depression (16%), and attention-deficit/hyperactivity disorder (9%).^53^ Health-related quality of life is substantially reduced in patients with PCOS, with body weight concerns, menstrual irregularity, and a sense of lack of control over health being important contributors.^52,71^ It is critical that PCOS in T2D is managed with a focus on biopsychosocial well-being to achieve positive health outcomes.

### Limitations

The limitations of this systematic review include that none of the studies had PCOS as a primary outcome. There was also a lack of a unified approach to diagnosing PCOS across studies and no reporting of the timing of menarche, which may have contributed to the high heterogeneity observed in the meta-analysis. Two of the largest studies did not report the criteria used for PCOS diagnosis.^45,46^ However, this systematic review had a comprehensive search strategy across several databases, including the gray literature, which includes all available evidence to date on this outcome.

The results of this study reflect the lack of consensus and difficulty in diagnosing PCOS in adolescents. The European Society of Human Reproduction and Embryology/American Society of Reproductive Medicine, the Pediatric Endocrine Society, and the International Consortium of Paediatric Endocrinology guidelines suggest that ultrasonography showing increased ovarian size could be used to aid in diagnosis, but other guidelines are more conservative in using these findings to diagnose PCOS.^14,15,16,17,18^ In addition, the European Society of Human Reproduction and Embryology/American Society of Reproductive Medicine guidelines state that biochemical hyperandrogenism needs to be present, and not just clinical signs of hyperandrogenism, whereas other guidelines state that either is sufficient for diagnosing PCOS.^14,15,16,17,18^ There is a need for a consensus to establish the pediatric criteria for diagnosing PCOS in adolescents to ensure accurate diagnosis and lower the misclassification rates.

Given these limitations, the results should be interpreted with caution. Larger multiethnic, longitudinal cohort studies evaluating PCOS prevalence in girls with T2D and using standardized criteria for defining PCOS are urgently needed.

## Conclusions

This study found that in girls with T2D, approximately 1 in 5 had PCOS. Identifying PCOS in this population is critical to allow for early screening and management of PCOS and its associated health concerns. Future studies are urgently needed to define the impact of obesity and race on PCOS prevalence in this population and to ensure the development of personalized assessment and treatment strategies.

## Notes

Supplement. **eTable 1.** Sample Search Strategy: MEDLINE **eTable 2.** Sample Search Strategy: Embase **eTable 3.** Search Strategy: CINAHL **eTable 4.** Search Strategy: Cochrane Library—Reviews and Trials **eTable 5.** Search Strategy: Web of Science—Conference Proceedings Citation Index–Science (CPCI- S), 1990-Present **eTable 6.** Summary of Guidelines for Diagnosing PCOS in Adolescents **eTable 7. **Risk of Bias of Included Studies
eReferences
