# Supplementary material for: Prevalence of Polycystic Ovary Syndrome in Patients With Pediatric Type 2 Diabetes: A Systematic Review and Meta-analysis
Source: JAMA Netw Open. 2022 Feb 15;5(2):e2147454. doi: 10.1001/jamanetworkopen.2021.47454 (PMC8848210; doi:10.1001/jamanetworkopen.2021.47454)
Supplement: Supplement. — eTable 1. Sample Search Strategy: MEDLINE eTable 2. Sample Search Strategy: Embase eTable 3. Search Strategy: CINAHL eTable 4. Search Strategy: Cochrane Library—Reviews and Trials eTable 5. Search Strategy: Web of Science—Conference Proceedings Citation Index–Science (CPCI- S), 1990-Present eTable 6. Summary of Guidelines for Diagnosing PCOS in Adolescents eTable 7. Risk of Bias of Included Studies eReferences [file jamanetwopen-e2147454-s001.pdf]

Supplemental Online Content

Cioana M, Deng J, Nadarajah A, et al. Prevalence of polycystic ovary syndrome in patients with pediatric type 2 diabetes: a systematic review and meta-analysis. *JAMA Netw Open*. 2022;5(2):e2147454. doi:10.1001/jamanetworkopen.2021.47454

eTable 1. Sample Search Strategy: MEDLINE
eTable 2. Sample Search Strategy: Embase
eTable 3. Search Strategy: CINAHL
eTable 4. Search Strategy: Cochrane Library—Reviews and Trials
eTable 5. Search Strategy: Web of Science—Conference Proceedings Citation Index-Science (CPCI-S), 1990-Present
eTable 6. Summary of Guidelines for Diagnosing PCOS in Adolescents
eTable 7. Risk of Bias of Included Studies
eReferences

This supplemental material has been provided by the authors to give readers additional information about their work.

© 2022 Cioana M et al. *JAMA Network Open.*

eTable 1. Sample Search Strategy: MEDLINE

1	exp Diabetes Mellitus, Type 2/
2	NIDDM.ti,ab,kf.
3	MODY.ti,ab,kf.
4	t2d*.ti,ab,kf.
5	((typ* two or typ?two or typ* 2 or typ* II or typ?2 or typ?II or typ* ii or typ?ii) adj4 diabet*).ti,ab,kf.
6	((non insulin or noninsulin or late or adult* or matur* or slow or stabl*) adj4 diabet*).ti,ab,kf.
7	((ketoresist* or keto* resist* or keto* prone) adj4 diabet*).ti,ab,kf.
8	or/1-7
9	exp Child/
10	child*.ti,ab,kf.
11	adolescen*.ti,ab,kf.
12	exp Adolescent/
13	youth*.ti,ab,kf.
14	teenage*.ti,ab,kf.
15	preadolescen*.ti,ab,kf.
16	Pediatrics/
17	p?ediatric*.ti,ab,kf.
18	pe?diatric*.ti,ab,kf.
19	or/9-18
20	8 and 19
21	Polycystic Ovary Syndrome/
22	(PCOS or PCOD).ti,ab,kf.
23	(ovar* adj3 (sclerocystic or polycystic or micropolycystic or degeneration)).ti,ab,kf.
24	or/21-23
25	20 and 24
26	Prevalence/
27	prevalence.ti,ab,kf.
28	prevalence studies/
29	Incidence/
30	incidence studies/
31	incidence.ti,ab,kf.
32	Epidemiology/
33	epidemiolog*.ti,ab,kf.
34	ep.fs.
35	epidemiologic methods/ or epidemiological monitoring/ or sentinel surveillance/
36	exp epidemiologic studies/
37	case-control.ti,ab,kf.
38	cohort.ti,ab,kf.

© 2022 Cioana M et al. *JAMA Network Open.*

39	prospective.ti,ab,kf.
40	longitudinal.ti,ab,kf.
41	retrospective.ti,ab,kf.
42	cross sectional.ti,ab,kf.
43	correlational.ti,ab,kf.
44	or/26-43
45	25 and 44
46	45 not (animals/ not (humans/ and animals/))
47	remove duplicates from 46

© 2022 Cioana M et al. *JAMA Network Open.*

eTable 2. Sample Search Strategy: Embase

1	non insulin dependent diabetes mellitus/
2	NIDDM.ti,ab,kw.
3	MODY.ti,ab,kw.
4	t2d*.ti,ab,kw.
5	((typ* two or typ?two or typ* 2 or typ* II or typ?2 or typ?II or typ* ii or typ?ii) adj4 diabet*).ti,ab,kw.
6	((non insulin or noninsulin or late or adult* or matur* or slow or stabl*) adj4 diabet*).ti,ab,kw.
7	((ketoresist* or keto* resist*) adj6 diabet*).ti,ab,kw.
8	or/1-7
9	exp child/
10	child*.ti,ab,kw.
11	adolescent/
12	adolescen*.ti,ab,kw.
13	youth*.ti,ab,kw.
14	teenage*.ti,ab,kw.
15	preadolescen*.ti,ab,kw.
16	pediatrics/
17	p?ediatric*.ti,ab,kw.
18	pe?diatric*.ti,ab,kw.
19	or/9-18
20	8 and 19
21	ovary polycystic disease/
22	(PCOS or PCOD).ti,ab,kw.
23	(ovar* adj3 (sclerocystic or polycystic or micropolycystic or degeneration)).ti,ab,kw.
24	or/21-23
25	20 and 24
26	prevalence/
27	prevalence.ti,ab,kw.
28	incidence/
29	incidence.ti,ab,kw.
30	epidemiology/
31	epidemiolog*.ti,ab,kw.
32	ep.fs.
33	epidemiological monitoring/
34	sentinel surveillance/
35	case-control.ti,ab,kw.
36	cohort.ti,ab,kw.
37	prospective.ti,ab,kw.
38	longitudinal.ti,ab,kw.

© 2022 Cioana M et al. *JAMA Network Open.*

39	retrospective.ti,ab,kw.
40	cross sectional.ti,ab,kw.
41	correlational.ti,ab,kw.
42	or/26-41
43	25 and 42
44	43 not (animals/ not (humans/ and animals/))
45	remove duplicates from 44

© 2022 Cioana M et al. *JAMA Network Open.*

eTable 3. Search Strategy: CINAHL

#	Query
S1	(MH "Child+")
S2	"child*"
S3	(MH "Adolescence+")
S4	"youth*"
S5	"teenage*"
S6	(MH "Pediatrics")
S7	"p?ediatric*"
S8	"p#ediatric*"
S9	"pe#diatric*"
S10	"pediatric*"
S11	"preadolescen*"
S12	S1 OR S2 OR S3 OR S4 OR S5 OR S6 OR S7 OR S8 OR S9 OR S10 OR S11
S13	(MH "Diabetes Mellitus, Type 2")
S14	"NIDDM"
S15	"MODY"
S16	"T2D*"
S17	(typ* two or typ?two or typ* 2 or typ* II or typ?2 or typ?II) N4 diabet*
S18	(non insulin or noninsulin or late or adult* or matur* or slow or stabl*) N4 diabet*
S19	(ketoresist* or keto* resist* or keto* prone) adj4 diabet*
S20	S13 OR S14 OR S15 OR S16 OR S17 OR S18 OR S19
S21	S12 AND S20
S22	(MH "Polycystic Ovary Syndrome")
S23	"PCOS" or "PCOD"
S24	ovar* N3 (sclerocystic or polycystic or micropolycystic or degeneration)
S25	S22 OR S23 OR S24
S26	(MH "Prevalence")
S27	"prevalence"
S28	(MH "Cross Sectional Studies")
S29	"cross section*"
S30	(MH "Incidence")
S31	"incidence"
S32	(MH "Epidemiology")
S33	"epidemiolog*"

© 2022 Cioana M et al. *JAMA Network Open.*

S34	(MH "Epidemiological Research")
S35	(MH "Prospective Studies") OR (MH "Cross Sectional Studies") OR (MH "Case Control Studies") OR (MH "Correlational Studies")
S36	"case control" or "cohort" or "prospective" or "retrospective" or "longitudinal" or "correlational"
S37	S26 OR S27 OR S28 OR S29 OR S30 OR S31 OR S32 OR S33 OR S34 OR S35 OR S36
S38	(S21 AND S25 AND S37) NOT (MH "Animals")

© 2022 Cioana M et al. *JAMA Network Open.*

eTable 4. Search Strategy: Cochrane Library—Reviews and Trials

child* OR youth* OR teenage* OR adolescen* OR pediatric* OR preadolescen* OR p?ediatric* OR pe?diatric* in Title Abstract Keyword	**AND**
NIDDM OR MODY OR t2d OR typ* two NEAR/4 diabet* OR typ?two NEAR/4 diabet* OR typ* 2 NEAR/4 diabet* OR typ* II NEAR/4 diabet* OR typ?2 NEAR/4 diabet* OR typ?II NEAR/4 diabet* OR typ* ii NEAR/4 diabet* OR typ?ii NEAR/4 diabet* OR non insulin NEAR/4 diabet* OR noninsulin NEAR/4 diabet* OR late or adult* NEAR/4 diabet* OR matur* NEAR/4 diabet* OR slow NEAR/4 diabet* OR stabl* NEAR/4 diabet* OR ketoresist* NEAR/4 diabet* OR keto* resist* NEAR/4 diabet* OR keto* prone NEAR/4 diabet* in Title Abstract Keyword	**AND**
PCOS OR PCOD OR ovar* NEAR/3 sclerocystic OR ovar* NEAR/3 polycystic OR ovar* NEAR/3 micropolycystic OR ovar* NEAR/3 degeneration in Title Abstract Keyword	
(Word variations have been searched)	

© 2022 Cioana M et al. *JAMA Network Open.*

eTable 5. Search Strategy: Web of Science—Conference Proceedings Citation Index–Science (CPCI-S), 1990-Present

#1	TI=(child* OR youth* OR teenage* OR adolescen* OR pediatric* OR preadolescen* OR p?ediatric* OR pe?diatric*)
#2	TS=(child* OR youth* OR teenage* OR adolescen* OR pediatric* OR preadolescen* OR p?ediatric* OR pe?diatric*)
#3	#1 OR #2
#4	TI=(NIDDM OR MODY OR t2d OR typ* two NEAR/4 diabet* OR typ?two NEAR/4 diabet* OR typ* 2 NEAR/4 diabet* OR typ* II NEAR/4 diabet* OR typ?2 NEAR/4 diabet* OR typ?II NEAR/4 diabet* OR typ* ii NEAR/4 diabet* OR typ?ii NEAR/4 diabet* OR non insulin NEAR/4 diabet* OR noninsulin NEAR/4 diabet* OR late or adult* NEAR/4 diabet* OR matur* NEAR/4 diabet* OR slow NEAR/4 diabet* OR stabl* NEAR/4 diabet* OR ketoresist* NEAR/4 diabet* OR keto* resist* NEAR/4 diabet* OR keto* prone NEAR/4 diabet*)
#5	TS=(NIDDM OR MODY OR t2d OR typ* two NEAR/4 diabet* OR typ?two NEAR/4 diabet* OR typ* 2 NEAR/4 diabet* OR typ* II NEAR/4 diabet* OR typ?2 NEAR/4 diabet* OR typ?II NEAR/4 diabet* OR typ* ii NEAR/4 diabet* OR typ?ii NEAR/4 diabet* OR non insulin NEAR/4 diabet* OR noninsulin NEAR/4 diabet* OR late or adult* NEAR/4 diabet* OR matur* NEAR/4 diabet* OR slow NEAR/4 diabet* OR stabl* NEAR/4 diabet* OR ketoresist* NEAR/4 diabet* OR keto* resist* NEAR/4 diabet* OR keto* prone NEAR/4 diabet*)
#6	#4 OR #5
#7	#3 AND #6
#8	TI=(PCOS OR PCOD OR ovar* NEAR/3 sclerocystic OR ovar* NEAR/3 polycystic OR ovar* NEAR/3 micropolycystic OR ovar* NEAR/3 degeneration)
#9	TS=(PCOS OR PCOD OR ovar* NEAR/3 sclerocystic OR ovar* NEAR/3 polycystic OR ovar* NEAR/3 micropolycystic OR ovar* NEAR/3 degeneration)
#10	#8 OR #9
#11	#7 AND #10

© 2022 Cioana M et al. *JAMA Network Open.*

eTable 6. Summary of Guidelines for Diagnosing PCOS in Adolescents

Guideline	PCOS Diagnostic Criteria
**European Society of Human Reproduction and Embryology/American Society of Reproductive Medicine Amsterdam PCOS Consensus Workshop[1] (2012)**	Required criteria:<br>1. Oligomenorrhea/amenorrhea<br>• Present for at least 2 years post menarche or primary amenorrhea at age 16 years<br>2. Hyperandrogenemia<br>• Elevated circulating androgens using sensitive assays rather than just signs of androgen excess<br>3. Ultrasound showing increased ovarian size (>10 cm^3)
**Endocrine Society Clinical Practice Guideline[2] (2013)**	Required criteria:<br>1. Persistent oligomenorrhea<br>• 2 years after menarche<br>2. Hyperandrogenism<br>• Clinical: hirsutism, acne AND/OR<br>• Biochemical: hyperandrogenism after exclusion of other pathologies<br>Comments:<br>1. Anovulatory symptoms and polycystic ovarian morphology on ultrasound are not sufficient to make a diagnosis in adolescents
**Pediatric Endocrine Society/Androgen Excess-PCOS Society/International Pediatric Endocrine Societies[3] (2015)**	Required criteria:<br>1. Oligo-anovulation<br>• Menstrual intervals persistently <20 days or >45 days in individuals 2 or more years after menarche, consecutive menstrual intervals >90 days, or lack of onset of menses by age 15 years or by more than 2-3 years after thelarche regardless of chronologic age<br>2. Hyperandrogenism<br>• Clinical: moderate to severe hirsutism; acne that is persistent and poorly responsive to topical dermatologic therapy should be evaluated for the presence of hyperandrogenemia before initiation of any medical therapies<br>• Biochemical: persistent elevation of serum total and/or free testosterone levels, determined in a reliable reference laboratory; a single androgen level >2 SD above the mean for the specific assay should not be considered evidence of hyperandrogenism in an otherwise asymptomatic adolescent girl<br><br>Optional:<br>1. Polycystic ovarian morphology<br>• Ovarian imaging can be deferred during the diagnostic evaluation for PCOS<br>• In healthy girls with regular menstrual cycles and without hyperandrogenism this does not indicate a PCOS diagnosis<br>• Ovarian volume >12 cm^3 (by formula for a prolate ellipsoid) can be considered enlarged, follicle counts should not be utilized to define polycystic ovarian morphology in adolescents, and a multifollicular pattern (presence of large follicles distributed throughout the ovary) should not be considered a pathological finding<br><br>Not recommended:<br>1. Insulin resistance<br>2. Hyperinsulinemia<br>3. Anti-Müllerian hormone levels

© 2022 Cioana M et al. *JAMA Network Open.*

**International Consortium of Paediatric Endocrinology[4] (2017)**	Required criteria:<br>**1.** Irregular menses/oligomenorrhea<br>• Menstrual cycles >45 days; persistence of oligomenorrhea, secondary amenorrhea (absence of cycles for more than 3 months), or primary amenorrhea in girls with completed puberty<br>**2.** Hyperandrogenism<br>• Clinical: moderate to severe hirsutism; isolated acne and/or alopecia should not be considered diagnostic; moderate or severe inflammatory acne unresponsive to topical therapy may require investigation of androgen excess<br>• Biochemical: total and/or free testosterone measured in a reliable reference laboratory<br>Optional:<br>1. Polycystic ovarian morphology: in an adolescent without hyperandrogenism/oligo-anovulation this does not indicate a PCOS diagnosis; measurement of ovarian volume, follicle number and size, and uterine dimensions may be useful in the evaluation of amenorrhea, but is not needed for PCOS diagnosis<br>2. Severe cystic acne<br><br>Not recommended as diagnostic markers:<br>1. Obesity<br>2. Insulin resistance<br>3. Hyperinsulinemia<br>4. Biomarkers (e.g. Anti-Müllerian hormone, Testosterone/ Dihydrotestosterone ratio)<br>5. Acanthosis nigricans<br><br>Comments:<br>1. Must generally be 2 years post-menarche<br>2. Must rule out other disorders of hyperandrogenism (e.g. non-classical congenital adrenal hyperplasia, Cushing's syndrome)
**International Evidence-Based Guideline for the Assessment and Management of PCOS[5] (2020)**	Required criteria:<br>1. Irregular menses<br>• >90 days for any one cycle (>1 year post-menarche)<br>• Cycles <21 or >45 days (>1 to <3 years post-menarche)<br>• Cycles <21 or >35 days or <8 cycles per year (>3 years post-menarche)<br>• Primary amenorrhea by age 15 or >3 years post-thelarche<br>• Irregular menstrual cycles (<1 year post-menarche) represent normal pubertal transition<br>2. Hyperandrogenism<br>• Clinical: hirsutism (modified Ferriman-Gallwey score recommended), severe acne, AND/OR<br>• Biochemical: hyperandrogenaemia confirmed using validated high-quality assays (free testosterone, free androgen index, or bioavailable testosterone)<br><br>Not recommended:<br>1. Pelvic ultrasound for diagnosis within 8 years post menarche<br>2. Anti-Müllerian hormone levels
**Common Diagnostic Criteria**	**1. Persistent oligomenorrhea**<br>**2. Clinical and/or biochemical hyperandrogenism**

© 2022 Cioana M et al. *JAMA Network Open.*

eTable 7. Risk of Bias of Included Studies

	External Validity Items					Internal Validity Items					Overall Score	Overall Risk of Bias
**Author, year**	**1**	**2**	**3**	**4**	**5**	**6**	**7**	**8**	**9**	**10**		
Amed et al,[6] 2012	1	0	0	1	1	0	0	1	1	1	6	moderate
Amutha et al,[7] 2012	0	1	1	1	1	0	0	1	1	1	7	moderate
Balasanthiran et al,[8] 2012	0	0	1	1	1	1	1	1	1	1	8	moderate
Pérez-Perdomo et al,[9] 2005	1	0	0	0	1	0	0	0	1	0	3	high
Shield et al,[10] 2009	1	1	1	1	1	1	1	1	1	1	10	low
Zdravkovic et al,[11] 2004	0	1	1	1	1	1	1	1	1	1	9	low

Footnote: 0: no, 1: yes, overall risk of bias: low (score >8), moderate (score 6-8), or high (score ≤5). Items scored: 1) Was the study's target population a close representation of the national population in relation to relevant variables, e.g., age, sex?; 2) Was the sampling frame a true or close representation of the target population?; 3) Was some form of random selection used to select the sample, OR, was a census undertaken?; 4) Was the likelihood of non-response bias minimal?; 5) Were data collected directly from the subjects (as opposed to a proxy)?; 6) Was an acceptable case definition used in the study?; 7) Had the study instrument that measured the parameter of interest (e.g., prevalence of comorbidity) been tested for reliability and validity (if necessary)?; 8) Was the same mode of data collection used for all subjects?; 9) Was the length of the shortest prevalence period for the parameter of interest appropriate?; 10) Were the numerator(s) and denominator(s) for the parameter of interest appropriate?

© 2022 Cioana M et al. *JAMA Network Open.*

eReferences.

1. Fauser BCJM, Tarlatzis BC, Rebar RW, et al. Consensus on women's health aspects of polycystic ovary syndrome (PCOS): the Amsterdam ESHRE/ASRM-Sponsored 3rd PCOS Consensus Workshop Group. *Fertil Steril*. 2012;97(1):28-38.e25. doi:10.1016/j.fertnstert.2011.09.024

2. Legro RS, Arslanian SA, Ehrmann DA, et al. Diagnosis and treatment of polycystic ovary syndrome: an Endocrine Society clinical practice guideline. *J Clin Endocrinol Metab*. 2013;98(12):4565-4592. doi:10.1210/jc.2013-2350

3. Witchel SF, Oberfield S, Rosenfield RL, et al. The Diagnosis of Polycystic Ovary Syndrome during Adolescence. *Horm Res Paediatr*. 2015;83:376-389. doi:10.1159/000375530

4. Ibáñez L, Oberfield SE, Witchel S, et al. An International Consortium Update: Pathophysiology, Diagnosis, and Treatment of Polycystic Ovarian Syndrome in Adolescence. *Horm Res Paediatr*. 2017;88(6):371-395. doi:10.1159/000479371

5. Peña AS, Witchel SF, Hoeger KM, et al. Adolescent polycystic ovary syndrome according to the international evidence-based guideline. *BMC Medicine*. 2020;18(1):72. doi:10.1186/s12916-020-01516-x

6. Amed S, Hamilton JK, Sellers EAC, et al. Differing clinical features in Aboriginal vs. non-Aboriginal children presenting with type 2 diabetes. *Pediatric Diabetes*. 2012;13(6):470-475. doi:10.1111/j.1399-5448.2012.00859.x

7. Amutha A, Datta M, Unnikrishnan R, Anjana RM, Mohan V. Clinical profile and complications of childhood- and adolescent-onset type 2 diabetes seen at a diabetes center in south India. *Diabetes Technol Ther*. 2012;14(6):497-504. doi:10.1089/dia.2011.0283

8. Balasanthiran A, O'Shea T, Moodambail A, et al. Type 2 diabetes in children and young adults in East London: an alarmingly high prevalence. *Practical Diabetes*. 2012;29(5):193-198a. doi:10.1002/pdi.1689

9. Pérez-Perdomo R, Pérez-Cardona CM, Allende-Vigo M, Rivera-Rodríguez MI, Rodríguez-Lugo LA. Type 2 diabetes mellitus among youth in Puerto Rico, 2003. *P R Health Sci J*. 2005;24(2):111-117.

10. Shield JPH, Lynn R, Wan KC, Haines L, Barrett TG. Management and 1 year outcome for UK children with type 2 diabetes. *Archives of Disease in Childhood*. 2009;94(3):206-209. doi:10.1136/adc.2008.143313

11. Zdravkovic V, Daneman D, Hamilton J. Presentation and course of Type 2 diabetes in youth in a large multi-ethnic city. *Diabetic Medicine*. 2004;21(10):1144-1148. doi:10.1111/j.1464-5491.2004.01297.x

© 2022 Cioana M et al. *JAMA Network Open.*
